# MIDAS: Multi-Sensorial Immersive Dynamic Autonomous System for Stroke Hand Rehabilitation-Prototype Design and Exploratory User Evaluation

**DOI:** 10.3390/s26206384

**Published:** 2026-10-09

**Authors:** Anoop Kumar Sinha, Fok-Chi-Seng Fok Kow, Zhang Jin Ming, Bao Songyu, Jake Tan Jun Kang, Hong Yan Jack Jeffrey, Galina Mihaleva, Nadia Magnenat Thalmann, Yiyu Cai

**Affiliations:** 1 School of Mechanical and Aerospace Engineering, Nanyang Technological University, 65 Nanyang Dr, Singapore 637460, Singapore; 2 Currently with the School of Science and Technology, Singapore University of Social Sciences, 463 Clementi Road, Singapore 599494, Singapore; 3 Institute for Media Innovation (IMI), Nanyang Technological University (NTU), Singapore 637553, Singapore; nadia.thalmann@unige.ch; 4 School of Art, Design and Media, Nanyang Technological University, 81 Nanyang Dr, Singapore 637458, Singapore; jason.fokkow@gmail.com (F.-C.-S.F.K.); zjm5252@gmail.com (Z.J.M.); songyu.bao00@gmail.com (B.S.); jake_tan@mde.harvard.edu (J.T.J.K.); jeffreyhong@ntu.edu.sg (H.Y.J.J.); galina.mihaleva@asu.edu (G.M.)

**Keywords:** rehabilitation, hand exoskeleton, virtual reality, immersive game, olfactory device, pilot study, user experience, self-rehabilitation motivation, 3D printing

## Abstract

Many stroke survivors experience impaired function in their paretic hands. Maintaining motivation to continue hand rehabilitation exercises can be challenging. This paper presents MIDAS (Multi-Sensorial Immersive Dynamic Autonomous System), a proof-of-concept immersive system developed to explore feasibility, user experience, and self-reported responses during hand rehabilitation after stroke. MIDAS is lightweight and portable. It consists of a hand exoskeleton subsystem, a virtual reality (VR) subsystem, and an olfactory subsystem. Together, these components engage four senses during rehabilitation. A pilot study involving five people affected by stroke was conducted across three sessions. The subsystems of MIDAS were added progressively in each session. The game environment, sound effects, and scent were selected to create a multisensory experience. User experience scores were higher in Session 3 than in Session 1 for four participants and unchanged for one. Stroke Rehabilitation Motivation Scale (SRMS) scores decreased across sessions for two participants, increased between Sessions 1 and 3 for two, and remained unchanged for one. Responses to several individual questionnaire items were favorable, but the study did not establish an overall increase in rehabilitation motivation.

## 1. Introduction

Stroke rehabilitation is a long and taxing process that requires commitment and determination from patients. Therefore, it is important to ensure that the rehabilitation process is stimulating. One key factor is motivation, which is an essential factor that contributes to exercise adherence and the outcome of stroke rehabilitation. Researchers distinguish between intrinsic and extrinsic motivation [1]. Intrinsic motivation refers to behavior that is driven by internal rewards [2]. On the other hand, extrinsic motivation refers to behavior that is driven by external rewards, such as money, fame, grades, and praise [3,4]. Most patients have extrinsic motivation during stroke rehabilitation. It is essential to increase their intrinsic motivation, which can be enhanced by design features of the rehabilitation task that the patient is performing [5].

Many laboratories have created exoskeletons and serious games that utilize bioengineering and robotics in autonomous or semi-autonomous systems for stroke rehabilitation. In [6], authors reported development of an electromyography (EMG)-controlled serious game for rehabilitation. In this study, subjects were instructed to control the cursor on a computer screen with their non-dominant hand. The BRAVO hand exoskeleton reported in [7] is an EMG-driven hand exoskeleton for grasping cylindrical objects. A distinctive feature of the BRAVO hand exoskeleton design is its intrinsic adaptability to the patient’s hand size. The BRAVO hand exoskeleton uses two electric actuators, one to cause flexion and extension of the thumb and the other for the remaining fingers of the subject’s hand. NREX [8] is a low-cost exoskeleton robot developed for upper limb rehabilitation from elbow to wrist. It uses two motorized axes for elbow and wrist joints. The authors claim that it can assist in reaching and drinking tasks. Another system by Hong Kong Polytechnic University [9] enables stroke-affected subjects to perform hand training at different intensities. The subject’s intention from the hemiplegic side is captured using surface electromyography (sEMG) signals. The exoskeleton hand robotic training then assists in hand opening or hand closing functional tasks. In [10], the authors presented a wearable exoskeleton. This exoskeleton is designed as an anterior-support type device to achieve palmar extension. The bend angle data extracted using a glove with strain gauges from the non-paretic hand is transferred to the exoskeleton on the paretic hand to mimic the movements of the non-paretic hand. HERCULES, a 3-degree-of-freedom (DoF) pneumatically operated exoskeleton arm for upper limb rehabilitation, is reported in [11]. The exoskeleton arm provides elbow flexion and extension, shoulder flexion and extension, and shoulder abduction and adduction. A semi-autonomous hybrid electroencephalography/electrooculography (EEG/EOG)-controlled whole-arm exoskeleton is presented in [12]. The whole-arm exoskeleton consisted of a shoulder–elbow module and wrist–hand module. The authors report that the new hybrid paradigm combining EEG and EOG is user-friendly. A limitation of most rehabilitation devices is non-portability. Authors in [13] suggest avoiding conventional materials for manufacturing that could make the robot bulky, expensive and difficult to transport.

Current efforts to increase motivation include creating video games [14,15], inventing interactive game-based rehabilitation tools [16,17,18,19], and inventing immersive escape room games [20,21]. Move-IT [22] is a game designed for upper limb stroke patients. It uses an Oculus Rift Head-Mounted Display (HMD) and a Leap Motion hand tracker. Authors in this study showed that subjects were pleased with the system after the session. It also motivated the subjects to perform rehabilitation exercises. A low-cost wearable device, integrated into VR environments for a better rehabilitation process in patients with motor disabilities, is presented in [23]. The device translates the movement of the subject’s arm into the virtual jigsaw puzzle environment. A comparison between VR technology and conventional therapy for physical rehabilitation among patients with neurological deficits is presented in [24]. In this study, the patients were divided into two groups. One group used a VR instrument called Medical Interactive Rehabilitation Assistant (MIRA) for rehabilitation, and the other group used traditional therapy. The intrinsic motivation of the patients was assessed by a 22-item task evaluation questionnaire. It was reported that the VR session had a constructive influence on patients and empowered patients to perform tasks autonomously. On the other hand, conventional exercises were boring, which decreased the motivation of patients for continuing treatment. In [25], authors established that integrating research in game design, motor learning, and neurophysiology with rehabilitation science can greatly improve the quality of rehabilitation. Since data collection for the present study in 2021, research on VR-assisted upper-limb rehabilitation after stroke has continued to develop. A 2022 systematic review and meta-analysis examined haptic gloves combined with semi-immersive VR [26], while a subsequent review examined fully immersive VR interventions [27]. A 2024 review found inconsistent effects on upper-limb function across trials combining robotic therapy with VR [28]. More recently, a randomized trial investigated a home-based VR hand exercise program over four weeks [29]. Together, these studies show the range of current approaches; their equipment, intervention duration, and outcome measures differ from those used in the present three-session feasibility study.

Previous work has used immersive head-mounted virtual reality for upper-limb rehabilitation after stroke, including Move-IT [22], while other approaches have linked wearable devices to virtual rehabilitation environments [23]. Here, *immersion* refers to the audiovisual experience provided by a head-mounted VR display, supplemented by physical hand assistance and game-linked scent cues. We developed the Multi-Sensorial Immersive Dynamic Autonomous System (MIDAS), a portable prototype integrating a hand exoskeleton triggered by forearm surface electromyography (sEMG), an audiovisual VR game, and olfactory feedback. The objective of this exploratory study was to describe the system and examine its feasibility, user experience, and participants’ self-reported motivation across three sessions involving five people affected by stroke. The engineering contribution lies in integrating these components within one prototype. Because the components were introduced in a fixed order and the sample was small, the findings cannot establish the independent effect of each component or clinical effectiveness. Neural plasticity and motor recovery mechanisms were not assessed.

## 2. Overview of Mechatronic Design of MIDAS

MIDAS consists of 3 subsystems, each of which engages one or more senses. The first subsystem is the hand exoskeleton that engages the patient’s sense of touch through the opening of the hand. Hand assistance was triggered using activity recorded from a selected forearm muscle by an sEMG sensor module. The second subsystem is the VR subsystem consisting of a VR headset (Oculus Quest 2), a VR game, and a VR controller device. This subsystem engages the user’s sense of sight and hearing. The last subsystem is the olfactory subsystem consisting of a face shield-cum-olfactory device and a circuit board. This subsystem engages the sense of smell. An overview of the design specifications of MIDAS is presented in Table 1.

### 2.1. The MIDAS System

The exoskeleton is the primary subsystem of the MIDAS as it not only performs the rehabilitation task but also acts as the central network that controls the other subsystems. It comprises two modules: the exoskeleton itself and sEMG sensors.

#### 2.1.1. The MIDAS Hand Exoskeleton Subsystem

While the essential requirement of an exoskeleton is to be able to actuate the stroke patient’s hand with a significant range of motion, works of literature and user feedback research further suggest that the device must be safe, comfortable, lightweight, easy to wear, and small [30]. These are necessary to deliver an unobtrusive user experience and to improve the patient’s motivation to use the device. These design objectives were kept in mind while proposing the design of the MIDAS exoskeleton. They were achieved by complying with anthropomorphic design, appropriate sizing of the components, and proper material selection for each component of the MIDAS exoskeleton.

**Figure 1:**
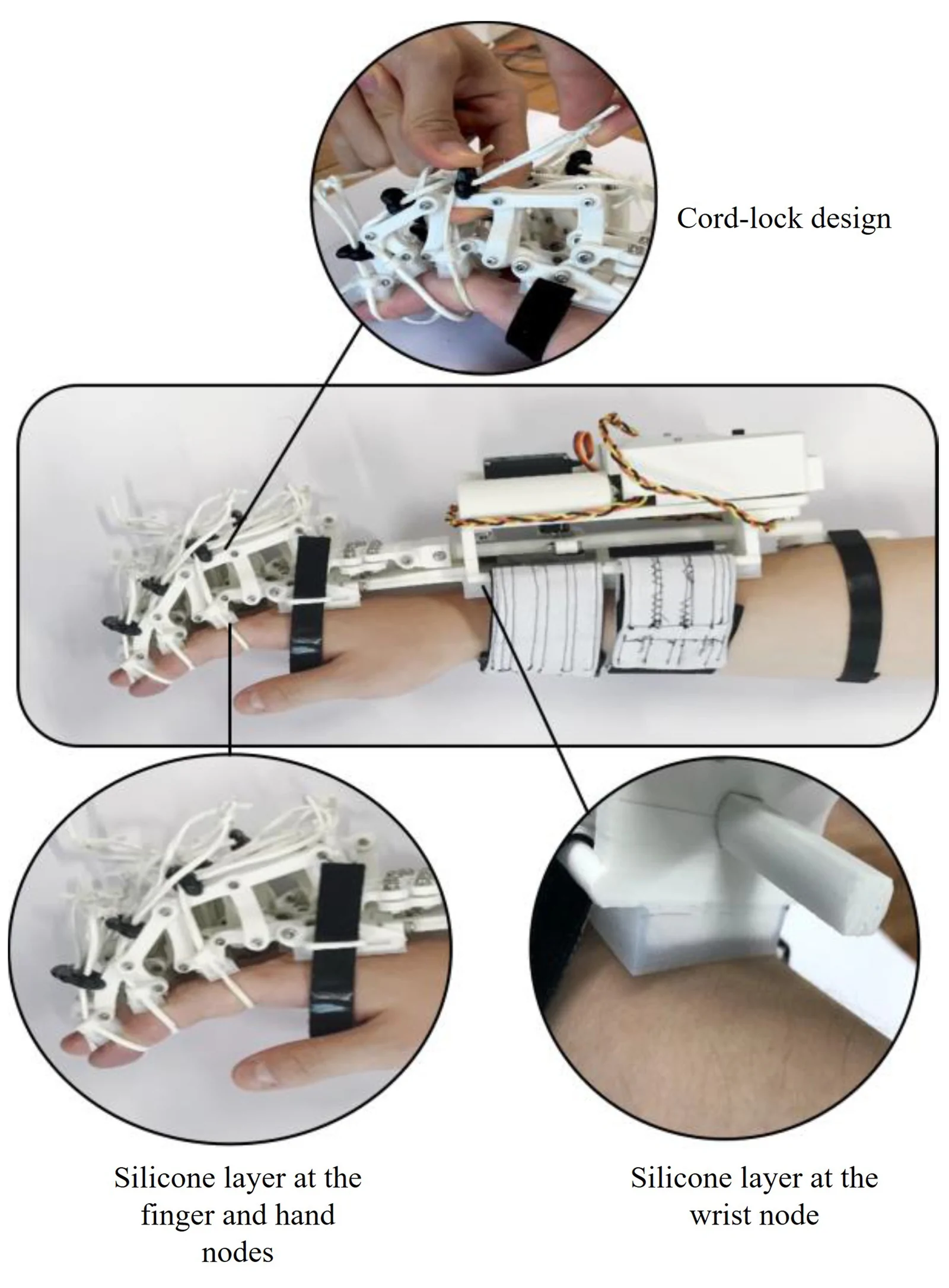
MIDAS exoskeleton.

The MIDAS exoskeleton is shown in Figure 1. The exoskeleton uses a linkage mechanism to provide flexion and extension of the patient’s fingers. The rotary motion of a servo motor (MG-996R) is translated into a shaft’s linear motion, which is then split four ways to actuate the mechanism on each finger. The rotary motion introduces interpolation into the linear movement, reducing sudden starts and stops, allowing MIDAS to be less obtrusive while aiding the patient. The single MG-996R servomotor drove the four-finger linkage during the study sessions, and all five participants used the exoskeleton as part of the study protocol. The servomotor’s specified stall torque is 9.4 kg cm at 5 V. Because fingertip force, joint torque, assisted range of motion under load, and finger flexor spasticity were not quantified, these observations do not establish the actuator’s capacity to extend hands with marked spasticity or fixed contracture. Future work should measure the forces delivered by the linkage and relate them to users’ hand impairment. The links connecting the actuation shaft to each finger mechanism were designed to permit two degrees of freedom, with the aim of accommodating differences in finger geometry and reducing constraints imposed by the linkage. These were design objectives rather than experimentally verified properties. Kinematic compatibility across different hand sizes, joint misalignment, and interaction forces were not quantified in this study; therefore, the two-DoF arrangement alone does not establish safe operation. In order to make the MIDAS hand exoskeleton compact and lightweight, we chose to actuate the fore, middle, ring, and little finger by using one servomotor. This makes the MIDAS hand exoskeleton compact and lightweight. In order to facilitate quick prototyping of the exoskeleton hand, which is essential to the development of the MIDAS, 3D printing with Polylactic acid (PLA) is preferred. PLA has a good strength-to-weight ratio, hence allowing for a design that is lightweight and compact. The total weight for the exoskeleton is around 748 g. Casted silicone pads are provided on the parts of the device in contact with the patient’s skin. These parts are, namely, the finger nodes, hand nodes, and wrist nodes. The soft material protects the patient’s skin from the rough surface of the 3D-printed parts and prolongs the duration the device can be worn. Silicone pads fabricated from Smooth-On Dragon-Skin 20 silicone are used because it is skin-safe and easily produced. Finally, attachments are provided to secure the exoskeleton on the patient’s hand. For the wrist and hand nodes, a double-sided Velcro strap design that wraps around the forearm and hand is used. A tab has been stitched at the end of each Velcro strap to prevent it from coming off the lug. Providing a tab also eliminates the need to slide the straps through the lug and allows a faster and easier securing process. For the finger nodes, a cord-lock design, explicitly developed for MIDAS, is used. The cord is tightened around the finger by using the cord-locks.

#### 2.1.2. The EMG Sensor Module

A non-invasive MyoWare surface electromyography (sEMG) sensor was used to record activity from a selected forearm muscle during attempted hand exercises. The two recording electrodes were positioned over the selected forearm muscle, and the reference electrode was placed over a bony area at the wrist. The system had two operating modes. In flexion mode, the recording electrodes were positioned over the flexor digitorum superficialis on the palmar side of the forearm to detect activity associated with finger flexion. The recorded activity was used as an input to trigger the corresponding exoskeleton movement. Only one muscle group was monitored in each operating mode, and simultaneous agonist–antagonist recordings were not available to assess co-contraction. Consequently, the recorded activity was not independently verified as voluntary movement intention, and the study did not establish the controller’s ability to reject involuntary or unintended activation.

The MyoWare sensor output was read by the microcontroller and smoothed using a 50-sample moving average before activation detection. This moving average reduces short-term fluctuations but does not by itself eliminate motion artifacts or other sources of EMG noise.

The retained implementation documentation does not establish whether the activation threshold was fixed or individualized, how it was calibrated, or whether additional temporal or control criteria were required before actuation. The precise triggering rule therefore cannot be reported retrospectively. This limits reproducibility of the sEMG control procedure and prevents assessment of its ability to distinguish task-related activation from unintended activation.

### 2.2. The MIDAS VR Subsystem

The VR subsystem of MIDAS includes an Oculus Quest 2 headset, a handheld VR controller device, and a VR game. The VR subsystem is intended to engage the user’s sense of sight and hearing. The environments in the game, indoor and outdoor, have been designed to allow the patient to feel relaxed and calm while playing the game. To accomplish this, natural locations such as the forest and the beach were chosen for the outdoor environments, while an earthy color palette was adopted for indoor environments to emulate nature. In line with the environment design direction, a toon shader was applied to all models to keep the game fun, simple, and casual.

**Figure 2:**
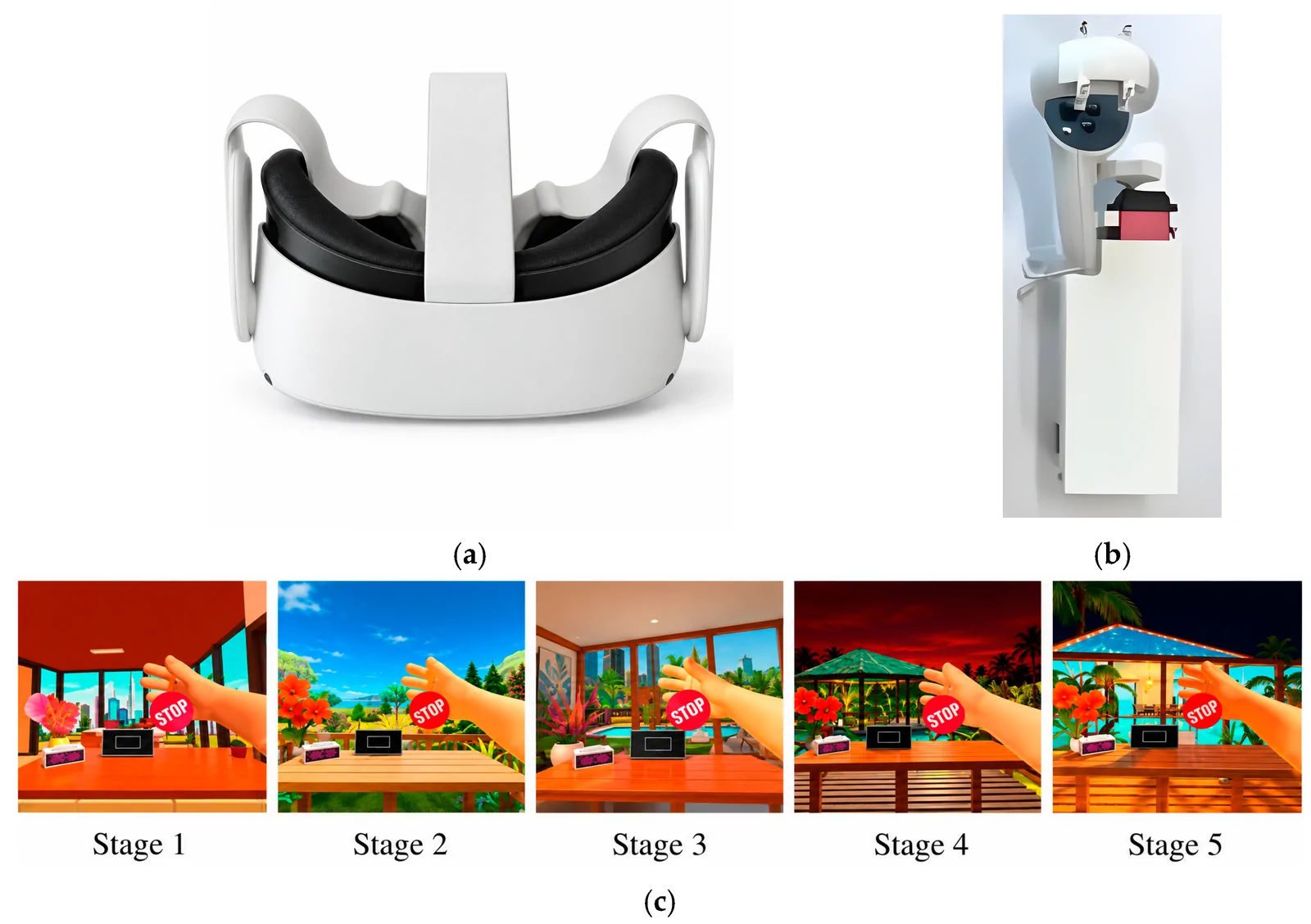
VR subsystem of MIDAS: (**a**) MIDAS Oculus Quest2 VR headset; (**b**) MIDAS handheld control device; (**c**) five stages of MIDAS VR game with different environments.

The game consists of five stages with different environments, each lasting 3 min. Figure 2 shows the VR headset, handheld controller, and game environments. When the participant attempted the target hand action, forearm sEMG activity was used to trigger the exoskeleton and a corresponding animation of the virtual hand. Control information was communicated to the VR subsystem via Bluetooth. The concurrent physical assistance and virtual-hand animation were intended to provide feedback linking recorded muscle activity to the displayed action. The rationale for combining muscle-activity-triggered assistance with visual feedback is supported by a previous stroke rehabilitation study that used EMG-triggered robotic assistance with real-time visual performance feedback and proprioceptive feedback [31]. That study provides a related design precedent; its clinical and neurophysiological findings cannot be extrapolated to MIDAS.

The mechanisms of the gameplay are designed to work for both types of stroke patients, i.e., patients with clenched hands (patients show finger flexion contractures) and patients who are stroke-affected, but fingers of the hand do not curl into themselves. The mechanism of the game is to score by squeezing the lemons. It is based on the duration of both the contraction and extension of the patient’s fingers. The number of squeezes increases as the game progresses. In each stage, as the cups are filled with lemonade, they change to become bigger variations of cups. Patients can visually associate their progress with familiar household objects and their real-world properties. This association allows for patients, who would typically not be exposed to VR technologies, to easily integrate into this reality and accept the environment and situation.

Game sound is also an essential feature for an immersive experience. Game sound, referring to all the sonic aspects of a game—ambient sounds, dialog, discrete sound effects, background music and interface sounds—has long been established as an indispensable part of modern video games [32,33]. It moves the narrative forward, conveys emotions and enriches the experience of the player. Game music has the potential to be much more than a passive element of the background. By tightly linking the content of gameplay and background music, the player becomes much more immersed in the gaming experience.

To provide game-related auditory cues to the participants, different layers of game sounds were incorporated into the game, including cheerful background music, bird chirping, the sound of a mouse character, the sound of squeezing a lemon and pentatonic scales-high-pitched chimes after completing a stage.

### 2.3. The MIDAS Olfactory Subsystem

Inhaled odorants are detected by receptors in the olfactory epithelium, and the resulting signals are transmitted to the brain [34]. Psychological responses to aroma inhalation have been investigated in specific settings, including a study of lavender inhalation and examination anxiety [35]. Olfactory stimuli have also been studied in relation to a range of psychological and physiological responses in humans, as reviewed by Angelucci et al. [36].

Olfactory feedback was integrated into MIDAS to add a scent cue to the VR game; its effects on mood or stress were not assessed in this study. A lemon and bergamot mixture was selected to accompany the lemon-squeezing task. In a study of healthy participants, inhalation of a lavender and bergamot mixture was associated with EEG changes compared with lavender alone [37]. Bergamot aromatherapy has also been studied in relation to work-related stress among teachers [38]. In a separate EEG study, lemon odor produced an immediate increase in theta activity that diminished over time [39]. Lemon was classified among fragrances considered to have sedative effects in a study of fragrance exposure during work tasks [40]. Another study reported improvements in anxiety and self-esteem after aromatherapy massage using a mixture of lavender, chamomile, rosemary, and lemon oils [41]. These studies involved different scents, delivery methods, and populations; they do not establish that the lemon and bergamot mixture used in MIDAS produced the same effects.

**Figure 3:**
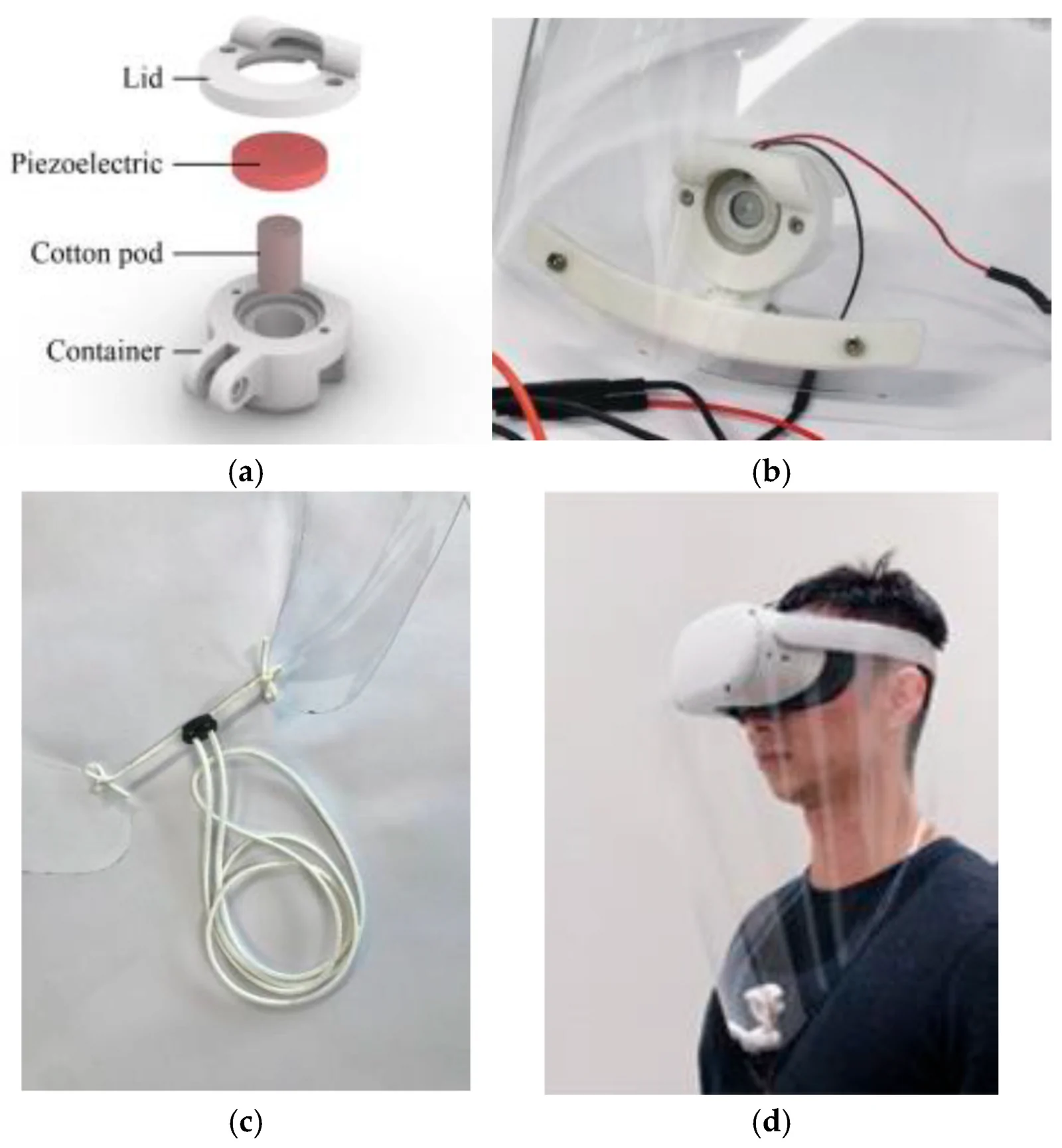
Olfactory subsystem of MIDAS: (**a**) exploded view of the olfactory device; (**b**) close-up view of the olfactory device; (**c**) cord attached to the end of face shield; (**d**) device worn on body.

The olfactory subsystem comprises two modules: a face shield-cum-olfactory device and a circuit board that connects the latter to the processing unit (see Figure 3). This subsystem works in conjunction with the exoskeleton and the VR subsystem by diffusing a lemon and bergamot scent when activated. In line with keeping a safe operation during COVID-19, the face shield is included, as patients will have their face masks removed to smell the scent. Figure 3a shows the exploded view of the main olfactory device. It consists of a 3D-printed lid and container holding in place a piezoelectric transducer and a cotton pod soaked in lemon and bergamot scent liquid. Alternating current (AC) voltage makes the piezoelectric transducer vibrate at a very high frequency and generates ultrasonic sound. The ultrasonic sound breaks the scented liquid into a fine mist, which is instantaneously distributed in the face shield. As for the face shield, a conventional Polyethylene Terephthalate (PET) frame is used. A cord on each end of the sheet is provided. When it is pulled, the PET frame forms a curve. To wear the device, the cords are passed through a cord lock to create a loop, which can then be put around the neck.

### 2.4. Control Scheme

**Figure 4:**
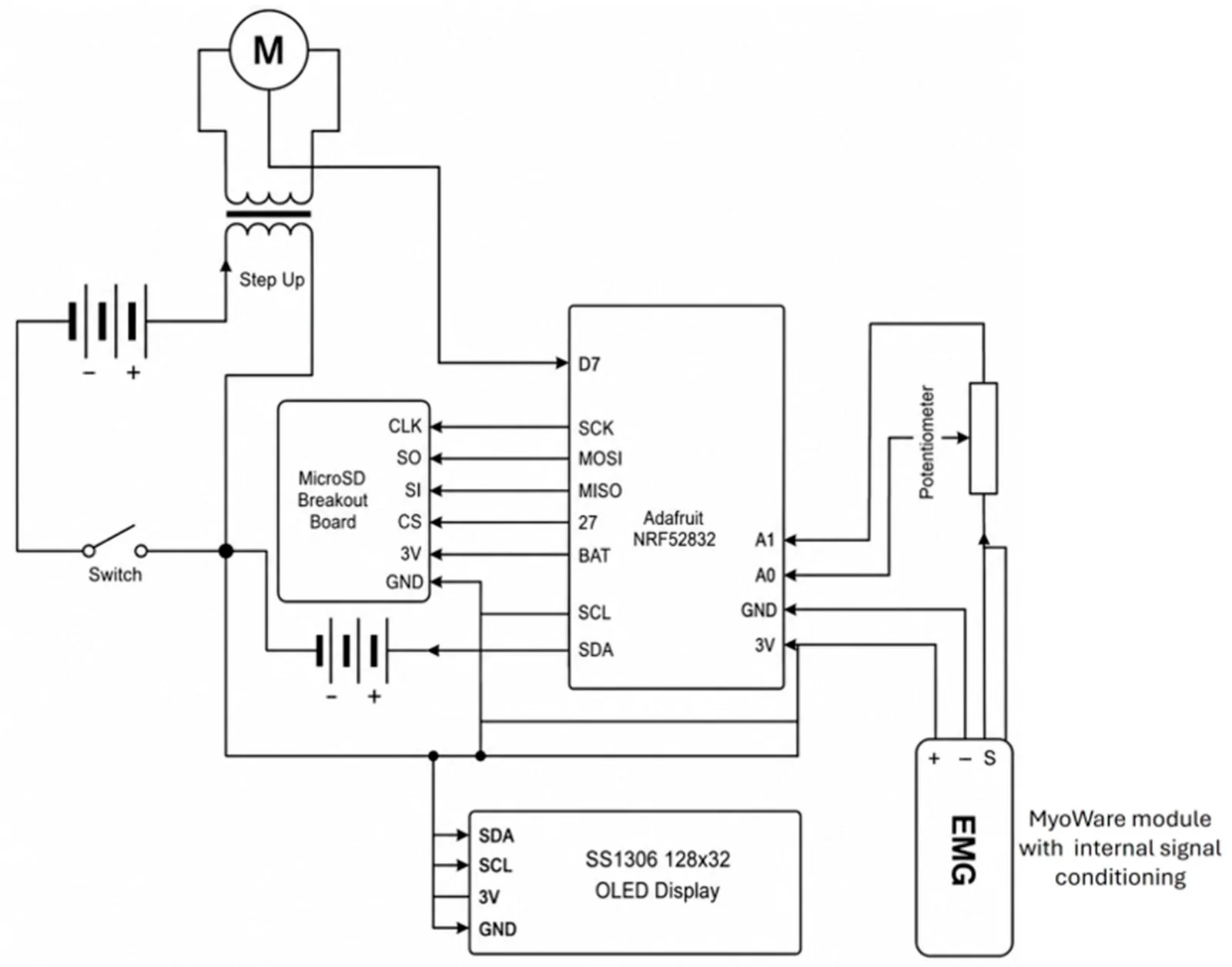
Circuitry behind MIDAS for communication between the EMG sensors and exoskeleton actuator.

**Figure 5:**
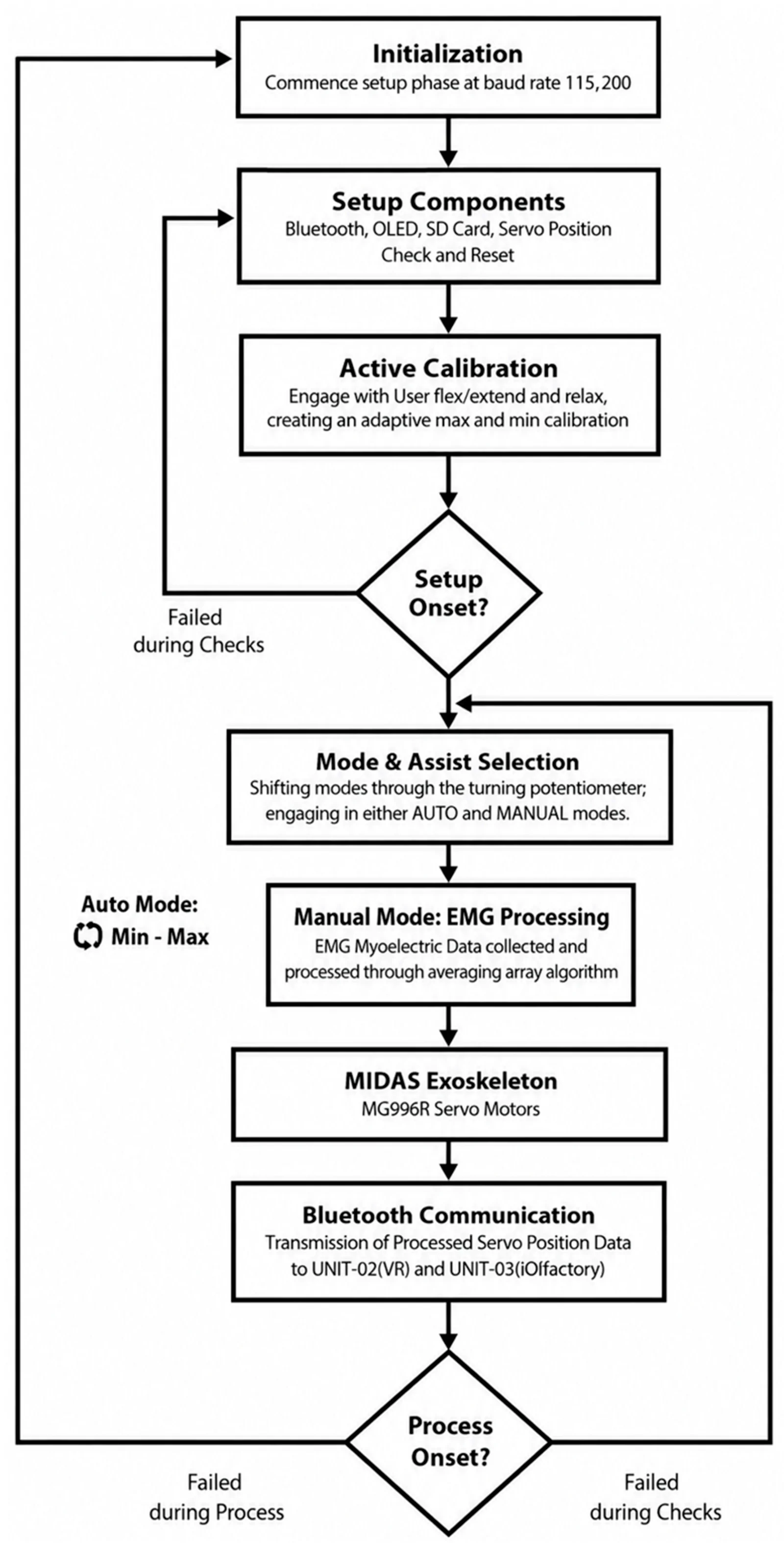
Flow chart of information processing in MIDAS.

The MIDAS circuit for communication between the hand exoskeleton and EMG sensors is shown in Figure 4. The chosen interface between the 3D-printed exoskeleton and the user was a commercially available microcontroller circuit board. In our case, the Adafruit Feather system was chosen due to its compact size and built-in functionalities. The circuitry runs on the Adafruit Feather nRF52832 microcontroller, SSD1306 128x32 OLED graphic display, MG-996R servo motor, MT3608 DC-DC voltage step-up module, Adafruit MicroSD SPI card breakout board, rotary potentiometer, an electrical switch and 2 sets of 2 Panasonic NCR18650B batteries. The electrodes were connected to the MyoWare sensor module, which contains analog signal-conditioning circuitry. The module amplifies and processes the small electrode signals before providing an analog output to the nRF52832 microcontroller. Thus, the microcontroller did not acquire unamplified electrode signals directly. The overall network diagram showing the channel for communication between different subsystems of MIDAS is shown in Figure 5. The MIDAS exoskeleton circuitry receives its commands from the EMG sensor module and relays this information to the microprocessor, where it will process the data by smoothing the sensor output with a 50-sample moving average, saving the data on the MicroSD card, sending the PWM data to the MG996 servo to actuate the exoskeleton, and sending the real-time data acquired to the VR subsystem and Olfactory subsystem. The microchip-based microcontroller (model Adafruit Feather NRF52832) is ARM-based, contains the latest Bluetooth technologies and is extremely energy efficient, all thanks to its single-chip architecture. By utilizing the latest nRF52832 MCU, it not only allows for faster and more efficient data transfer between Bluetooth peripherals but also allows the microcontroller to behave as a central connection between multiple other Bluetooth peripherals. This central hub mode is still in its early stages of development but has since made this project possible with almost no external networking peripherals. The effective sEMG acquisition frequency and exact mapping from detected muscle activity to servomotor commands could not be verified from the retained implementation records. Together with the undocumented activation criteria described in Section 2.1.2, these gaps limit reproducibility and assessment of trigger reliability.

## 3. Protocols and Tools for Pilot Study of MIDAS

Since this is a preliminary test, only 5 post-stroke patients were recruited to test the functionality and applicability of the device. The post-stroke patients were chosen according to the following criteria:

1. Age from 21 to 65.
2. Had a documented history of stroke and was considered eligible for the hand exercise sessions during study screening. No standardized stroke severity score or impairment threshold was recorded.
3. Passed the rapid cognitive screening (rapid cognitive screen score > 7).

### 3.1. Recruitment

The research team put up posters at local stroke rehabilitation organizations. Interested participants contacted the research team for participation. Consent was obtained after the researchers conducted screening tests and briefly explained the study objectives to patients. The process of data collection was carried out from the end of February 2021 to the end of March 2021 at the participant’s house or a place agreed upon by both the researchers and the participant.

Of the five participants, three were male, three were married, and four had received tertiary education. Three had left-sided impairment and two had right-sided impairment. Ages ranged from 27 to 61 years. Time since stroke onset ranged from 17 to 162 months (median: 38 months), indicating substantial variation in stroke chronicity. Participant characteristics are presented in Table 2. No standardized stroke severity classification was recorded. Motor performance was characterized using the Motor Assessment Scale, as reported in Section 5.1. However, the overall MAS score is not a hand-specific impairment measure. The available data therefore provide limited characterization of the upper-limb and hand impairments relevant to exoskeleton use.

### 3.2. Tools of Data Collection

#### 3.2.1. Structured Interviewing Questionnaire Form

The questionnaire includes socio-demographic characteristics (i.e., age, sex, marital status, educational level and occupation) and medical diagnosis.

#### 3.2.2. Motor Assessment Scale (MAS)

To evaluate motor function in recovering stroke patients, the MAS presented in [42] is used in this study. The MAS uses tasks related to activities of daily living to measure the full range of functional motor performance in stroke survivors. These consist of 9 items, which are supine to side lying, supine to sitting over the side of the bed, balanced sitting, sitting to standing, walking, upper-arm function, hand movements, advanced hand activities, and general tonus. Items (with the exception of the general tonus item) are assessed using a 7-point scale (0 to 6). A score of 6 indicates optimal motor behavior. Item scores (with the exception of the general tonus item) are summed to provide an overall score (out of 48 points). For the general tonus item, the score is based on continuous observations throughout the assessment. A score of 4 on this item indicates a consistently normal response; a score less than 4 indicates persistent hypertonus; and a score greater than 4 indicates various degrees of hypotonus.

#### 3.2.3. Montreal Cognitive Assessment (MoCA)

MoCA is developed as a rapid screening instrument for mild cognitive dysfunction [43]. It assesses different cognitive domains: attention and concentration, executive functions, memory, language, visuoconstructional skills, conceptual thinking, calculations, and orientation. It consists of 7 parts: visuospatial/executive, naming, memory, attention, language, abstraction, delayed recall, and orientation.

The maximum score for this tool is 30 points. A final total score of 26 and above is considered normal. One point is added for an individual who has 12 years or fewer of formal education.

#### 3.2.4. WHOQOL-BREF

It is an abbreviated version of the WHOQOL-100, which was developed by the WHO- QOL Group in 1994 [44]. It is used to cross-culturally assess quality of life. It consists of 4 domains: physical health, psychological, social relationships, and environment. It is a generic instrument to assess the positive and negative aspects of quality of life. The questions relating to the 4 facets, i.e., physical pain and discomfort experienced, psychological positive or negative feelings about life, social relationships such as companionship, love, and support desired in the personal relationship, and environmental factors such as the patient’s financial resources to live a healthy and comfortable life, were asked. The raw scores from the questions in these domains were then assigned.

#### 3.2.5. User Experience Questionnaire

To evaluate the user experience of MIDAS in this study, an adapted version of the original user experience questionnaire reported in [45] is used. It contains 8 items, namely, obstructive or supportive, complicated or easy, inefficient or efficient, clear or confusing, boring or exciting, non-interesting or interesting, conventional or inventive, and usual or leading edge. Each item is rated on a 7-point Likert scale. Table 3 shows the scoring system for the user experience questionnaire. The eight item scores were summed to obtain an overall score ranging from 8 to 56, with higher scores indicating more positive ratings. No validated interpretive cutoff was applied to this adapted total score.

#### 3.2.6. Stroke Rehabilitation Motivation Scale (SRMS)

The original SRMS, which consisted of 28 questions, was presented in [46]. It is used to measure internal and external factors influencing motivation among stroke patients to rehabilitate. A shorter version was also developed by the original creators of the scale. It comprises 7 questions that are correlated with the original 28 questions from the original SRMS. For this study, the shorter version is adapted, and the 7 questions are:

1. If you have choices, do you feel like choosing to do rehabilitation using our device?
2. Do you feel like your motivation for participating in rehabilitation has gotten higher after trying our device?
3. Do you feel the device could be useful in helping you to exercise your hand?
4. Do you want to participate in rehabilitation with our device if you see other stroke patients getting better through rehabilitation?
5. Would you perform rehabilitation with our device even if no one is enforcing it on you?
6. Do you find participating in rehabilitation with our device exciting?
7. Does doing rehabilitation with our device help you feel like you are achieving something?
8. Each item is rated on a 5-point Likert scale. A score of 1 = completely disagree to a score of 5 = completely agree. The score ranges from 7 to 35. A higher score means higher motivation.

#### 3.2.7. Self-Assessment Manikin (SAM)

SAM has been devised to assess the emotional response of a person to an object or event. Although there is ongoing debate regarding what defining features should be considered to capture a person’s emotions, authors in [47] have presented three dimensions, namely, pleasure, arousal, and dominance, associated with a person’s affective reaction to a stimulus to measure a person’s emotions. In this study, a 5-point scale is used to measure a person’s emotional response to MIDAS. Table 4 shows the scoring system for this tool.

## 4. Method of Data Collection and Analysis

**Figure 6:**
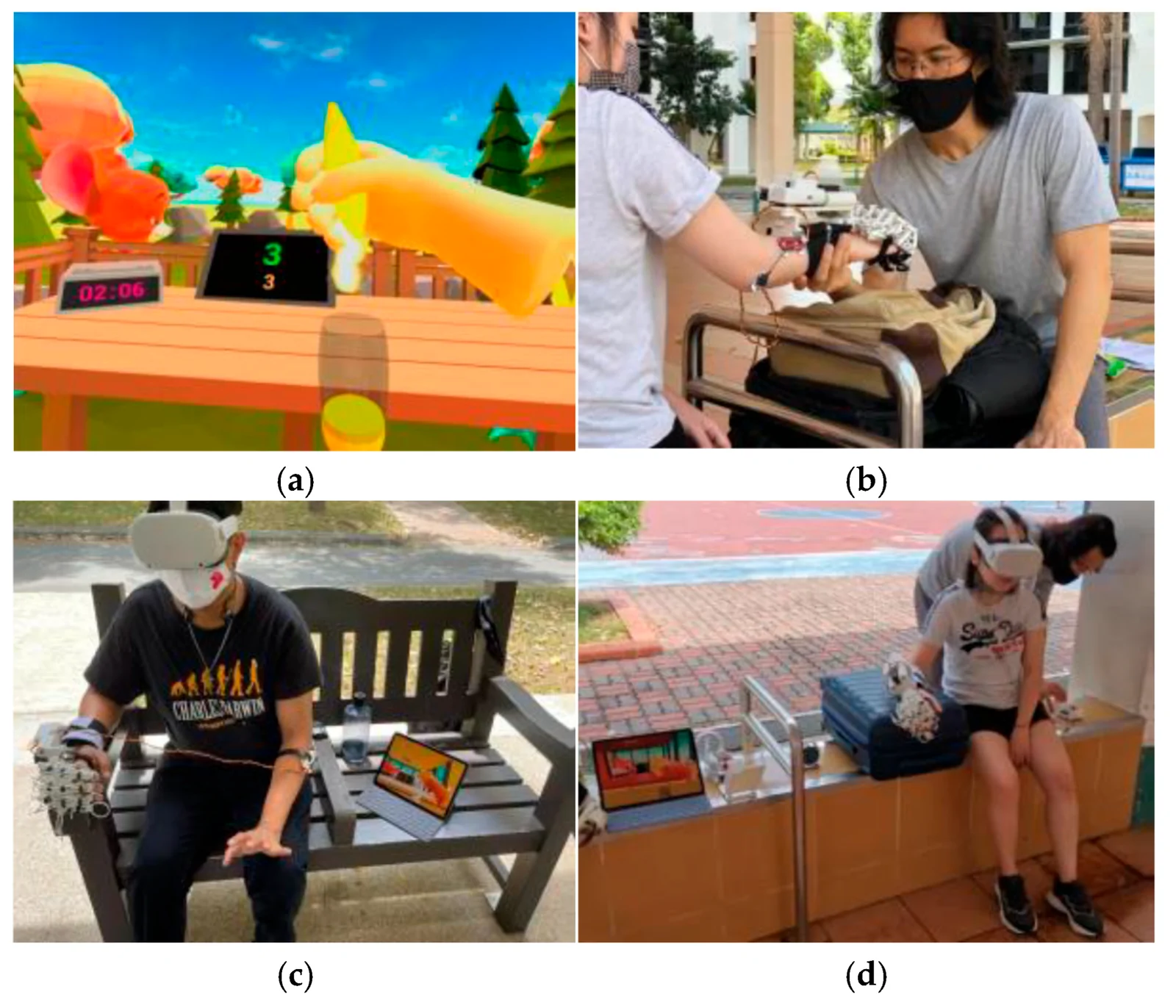
VR game environment and pilot study of MIDAS during the three sessions: (**a**) VR game showing the lemon squeezed to fill a cup; (**b**) patient using hand exoskeleton during Session 1; (**c**) patient using hand exoskeleton and VR during Session 2; (**d**) patient using hand exoskeleton, VR, and olfactory device during Session 3.

The data for the pilot study of MIDAS was collected in 3 sessions. Sessions were scheduled approximately one week apart, so the interval between Sessions 1 and 3 was approximately two weeks. The duration of each complete session was not recorded and could not be verified from the retained study documentation. The three subsystems of MIDAS were included in the study progressively in each session, one by one. In the 1st session, the patients were asked to wear the hand exoskeleton with the Myoware sEMG Sensors. The patient’s response to using the device was then recorded. The second session included recording the patient’s response when the hand exoskeleton, along with the VR subsystem, was used. In the third session, all the subsystems of MIDAS, i.e., the hand exoskeleton, the VR, and the olfactory subsystem, were used, and the patient’s response was recorded. This was done to evaluate the performance of MIDAS in engaging one or more senses of the patients in stages and its acceptability by the patients. In the 2nd and 3rd session, the patients were asked to play the VR game. Figure 6a shows the VR game and Figure 6b–d show the patients using MIDAS subsystems in the 3 different sessions.

Given the exploratory sample of five participants, individual trajectories and descriptive counts were the primary means of reporting results. As exploratory comparisons, Session 1 and Session 3 user experience and SRMS scores were evaluated using two-sided exact paired sign tests. Unchanged scores were excluded from these tests. The resulting *p* values were interpreted cautiously because of the small sample, multiple outcomes, and fixed order of the session conditions. No test was used to attribute a difference to an individual MIDAS component.

## 5. Results

In this section, the results of the pilot study of MIDAS are presented. The results of this exploratory study are presented descriptively. Individual scores and response patterns are reported to characterize participants’ use of MIDAS across the three sessions. Changes between sessions are not interpreted as evidence of clinical effectiveness because the sample was small and the system components were introduced in a fixed order.

### 5.1. MAS, MoCA, and QOL

**Figure 7:**
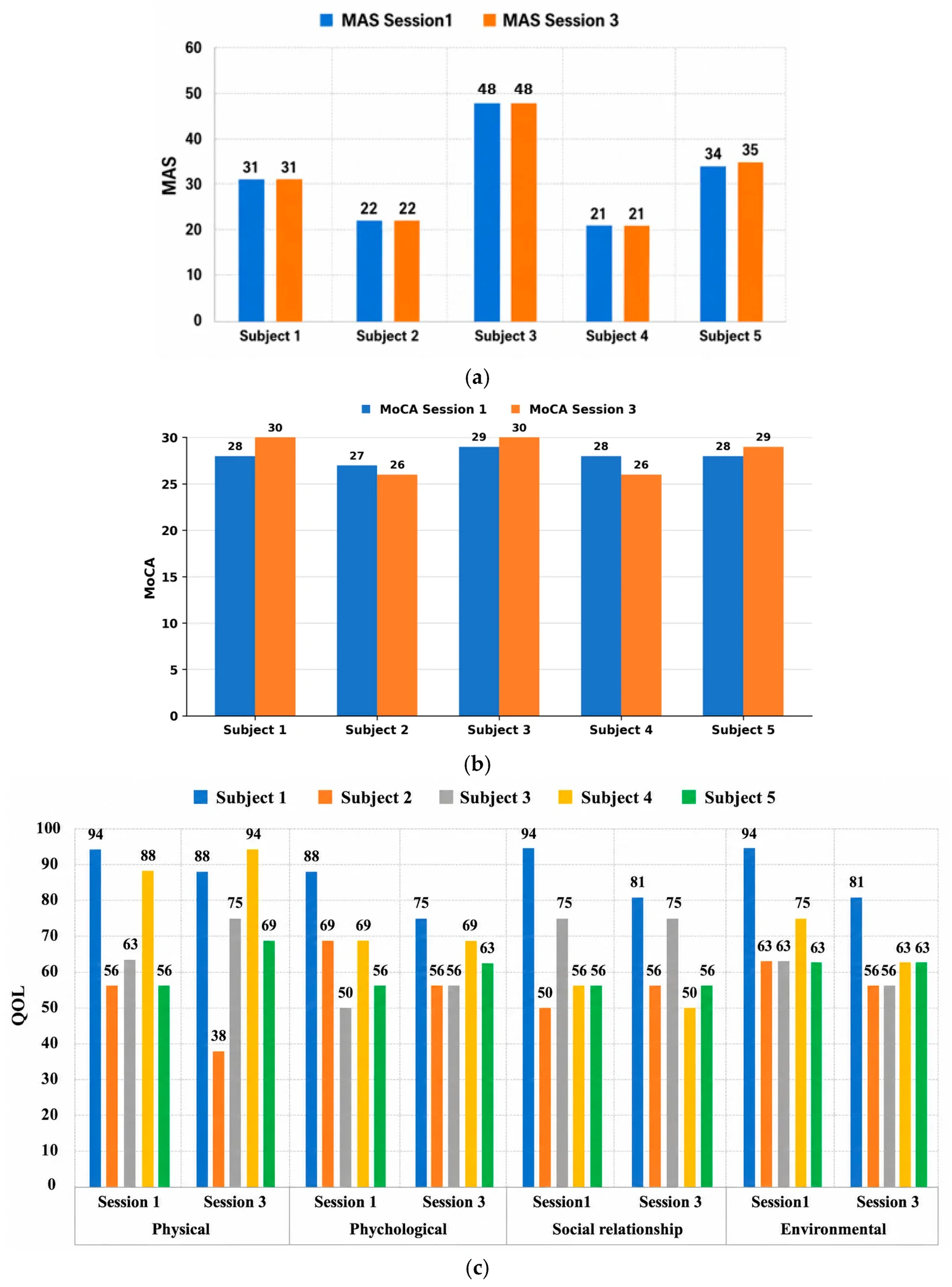
MAS, MoCA, and QOL scores of all subjects considered in this pilot study for Session 1 and Session 3: (**a**) MAS score of subjects for Session 1 and Session 3; (**b**) MoCA score of subjects for Session 1 and Session3; (**c**) QOL score of subjects for Session 1 and Session 3.

MAS and MoCA scores were used to describe participants’ motor and cognitive status during this exploratory study. These assessments were not intended to measure the effect of MIDAS over three sessions. Figure 7 presents individual MAS, MoCA, and quality-of-life (QOL) scores for Sessions 1 and 3. MAS scores varied among participants, and MoCA scores ranged from 26 to 30 across the two assessments. Individual MoCA scores changed by only a small number of points and in different directions between Sessions 1 and 3. Given the brief observation period and the absence of a control group, these changes were not interpreted as clinically meaningful cognitive improvement or decline attributable to MIDAS. QOL scores also varied: the lowest and highest values shown were 38 and 94 for physical health, 50 and 88 for psychological health, 50 and 94 for social relationships, and 56 and 94 for the environmental domain. Given the short observation period, small sample, and absence of a control group, differences between sessions cannot be interpreted as motor recovery, cognitive improvement, or changes in quality of life attributable to MIDAS.

### 5.2. User Experience Feedback

**Figure 8:**
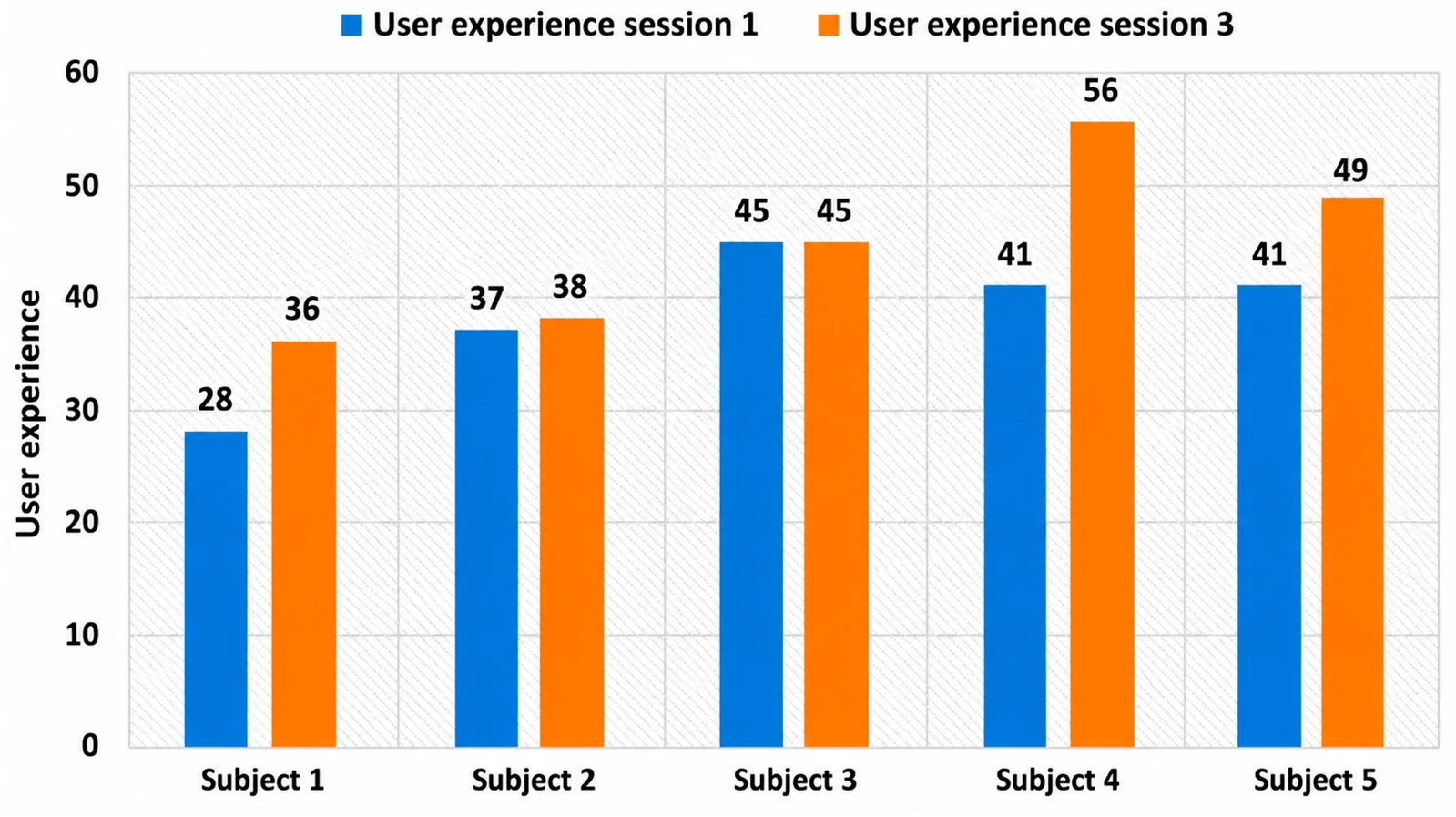
User experience feedback of subjects for Session 1 and Session 3.

Figure 8 shows the overall user experience scores given by each participant in Sessions 1 and 3. Table 5 presents their ratings for individual questionnaire items. Four participants had higher overall scores in Session 3, while one had the same score in both sessions. Overall scores ranged from 28 to 45 in Session 1 and from 36 to 56 in Session 3, on the adapted questionnaire’s possible range of 8–56. No validated cutoff was applied to classify overall user experience as favorable or unfavorable. For the “complicated–easy” item, four participants selected a rating toward the complicated end of the scale in Session 1, compared with one participant in Session 3. For the “conventional–inventive” item, one participant selected the highest rating in Session 1, compared with three participants in Session 3. These are descriptive observations; the study design cannot determine whether the differences in ratings were related to familiarity with the device, the VR subsystem, the olfactory subsystem, or other factors. Four participants had higher user experience scores in Session 3, and one had an unchanged score. An exploratory exact paired sign test did not provide evidence of a difference between Sessions 1 and 3 (*p* = 0.125).

### 5.3. Self-Rehabilitation Motivation of the Subjects

**Figure 9:**
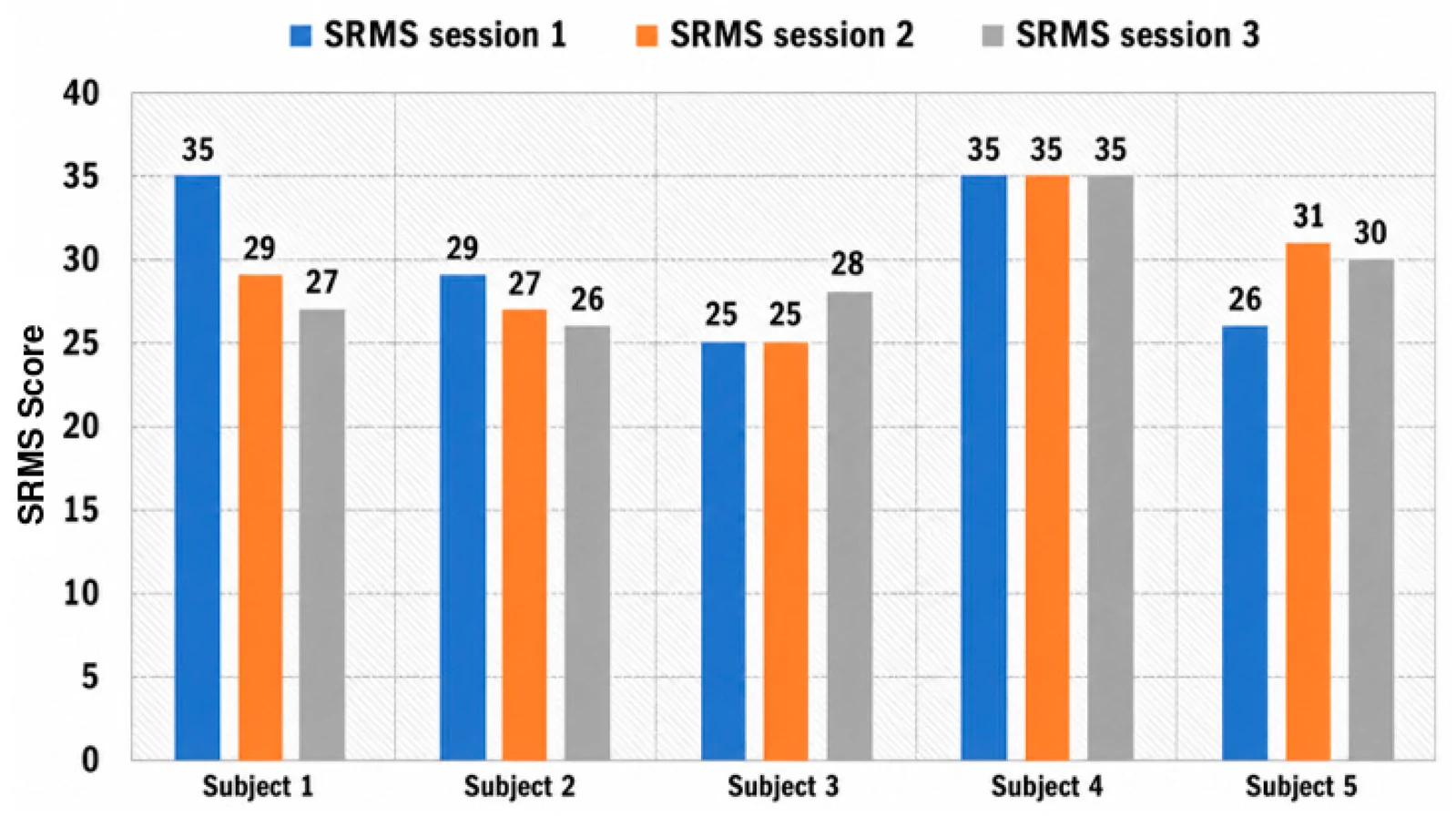
SRMS score of subjects for all sessions.

Figure 9 shows each participant’s Stroke Rehabilitation Motivation Scale (SRMS) score across the three sessions. The patterns differed among participants: scores decreased across sessions for two participants, increased from Session 1 to Session 3 for two participants, and remained unchanged for one participant. These mixed patterns do not establish an overall increase in rehabilitation motivation. Comparing Session 1 with Session 3, two participants had higher SRMS scores, two had lower scores, and one had an unchanged score. An exploratory exact paired sign test did not provide evidence of a consistent direction of change (*p* = 1.000).

Table 6 presents responses to the seven questionnaire items. Each item has 15 responses because the five participants answered it in each of the three sessions. All 15 responses to the question about choosing rehabilitation with the device were positive. A total of 11 of 15 responses to the question about increased motivation were positive; 12 of 15 responses indicated that the device could be useful for hand exercise; 14 of 15 responses indicated willingness to use it without enforcement; 13 of 15 responses rated participation with it as exciting; and 9 of 15 responses indicated a sense of achievement. These responses indicate favorable stated willingness to use the device at the times assessed. Because Table 6 pools responses across sessions, it does not establish that willingness increased for each participant between Sessions 1 and 3. These pooled item responses describe participants’ perceptions across sessions. They should not be interpreted as evidence that motivation increased over time, particularly because two participants’ total SRMS scores declined.

### 5.4. SAM

**Figure 10:**
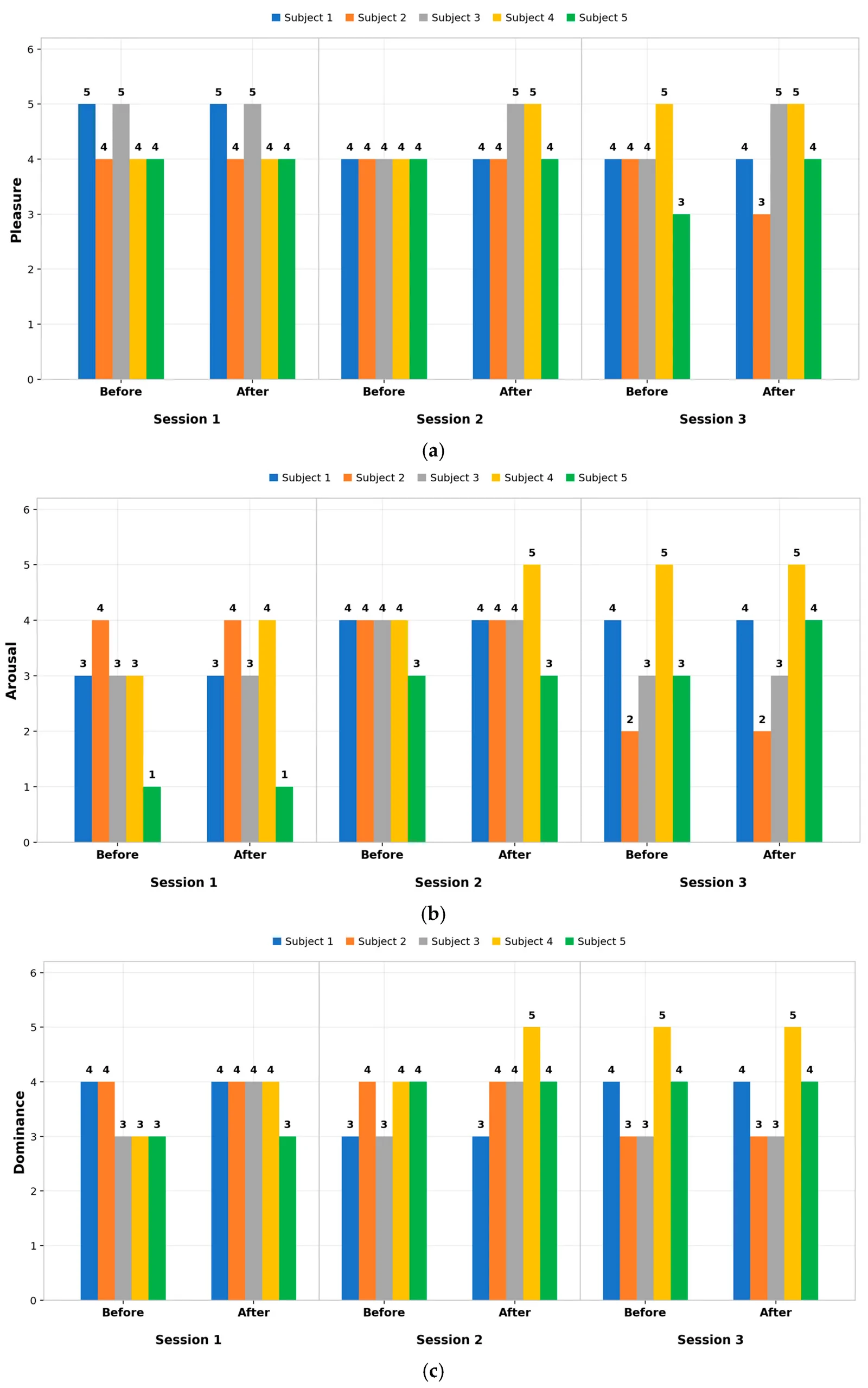
SAM scores of subjects for Session 1, Session 2 and Session 3: (**a**) difference in pleasure response of the subjects before and after using MIDAS during the three sessions; (**b**) difference in arousal response of the subjects before and after using MIDAS during the three sessions; (**c**) difference in dominance response of the subjects before and after using MIDAS during the three sessions.

Figure 10 shows participants’ Self-Assessment Manikin (SAM) ratings before and after device use in each session. Pleasure ratings were unchanged after device use in Session 1. In Session 2, pleasure ratings increased for two participants. In Session 3, pleasure ratings increased for two participants, decreased for one, and remained unchanged for two. Arousal ratings increased after device use for one participant in each of Sessions 1, 2, and 3; the remaining participants’ ratings were unchanged within those sessions. Dominance ratings increased for one participant in Session 1 and two participants in Session 2, while no participant’s rating changed in Session 3. These participant-level observations do not establish a consistent emotional effect of MIDAS.

## 6. Conclusions

This paper presented the Multi-Sensorial Immersive Dynamic Autonomous System (MIDAS), a portable prototype developed for assisted hand exercises after stroke. MIDAS integrates three subsystems: a hand exoskeleton triggered by forearm surface electromyography, an audiovisual VR game, and a device that releases scent in response to events in the game. Together, these components provide visual, auditory, tactile, and olfactory cues during exercise. Many of the prototype’s structural components were 3D printed, which may facilitate their modification for subsequent research. Its portability allowed the pilot sessions to be conducted at participants’ homes or other agreed locations.

The game used natural settings, music, and sound effects to create an audiovisual exercise environment. During the lemon-squeezing task, the olfactory subsystem was designed to release a lemon and bergamot scent. The purpose of combining these features was to explore an integrated, multisensory approach to assisted hand exercise. The present study did not isolate the contribution of any individual component or assess whether the scent changed participants’ mood or stress.

Five people affected by stroke participated in three exploratory sessions. The user experience results were generally favorable: four participants gave higher overall scores in Session 3 than in Session 1, while one gave the same score. Responses to several questionnaire items also indicated that participants viewed the device positively. However, self-reported motivation did not follow a uniform pattern. SRMS scores declined across sessions for two participants, increased from Session 1 to Session 3 for two, and remained unchanged for one. These mixed findings do not establish that MIDAS increased rehabilitation motivation. Likewise, changes in MAS, MoCA, or quality-of-life scores over the brief study period cannot be interpreted as evidence of clinical improvement.

Recent studies have examined haptic gloves combined with semi-immersive VR [26], fully immersive VR for upper-limb rehabilitation [27], robotic therapy combined with VR [28], and home-based VR hand exercise [29]. MIDAS integrates an sEMG-triggered hand exoskeleton, head-mounted VR, and a game-linked scent cue. However, these studies used different interventions, treatment durations, and outcome measures. The present three-session study describes participants’ experience with the prototype; it cannot establish the functional benefits evaluated in longer or controlled rehabilitation studies.

The hand exoskeleton is an important area for further engineering evaluation. The force delivered to each finger and the device’s ability to assist extension against different levels of spastic resistance remain to be measured. Measurements of finger force, movement range, comfort, and control reliability would help establish which users the current actuator and linkage can assist. If those measurements show that greater assistance is needed, future designs could evaluate a different actuator, an appropriate power supply, and structural materials selected through mechanical testing. Increasing motor torque alone would not establish that the device can safely assist a wider range of users.

The olfactory subsystem also offers scope for refinement. In the current prototype, an audible click occurs around scent release and may distract some users. A quieter switching arrangement could be evaluated in future versions. Additional game tasks and corresponding scent cues could also be explored, alongside measures of whether users detect the cues and find them acceptable.

This study has several limitations. It involved five participants and three sessions, with the exoskeleton, VR, and olfactory components introduced in a fixed order. Participants also differed considerably in time since stroke onset (17–162 months), and stroke severity was not documented using a standardized measure. This variation limits interpretation of differences between participants and the applicability of the findings to a defined impairment group. The design therefore cannot distinguish the effects of added components from familiarity with the system or other effects of repeated participation. In addition, the retained implementation documentation does not fully establish the sEMG acquisition frequency, activation-threshold calibration, or the exact mapping of detected activity to servomotor commands, limiting assessment of control reproducibility. Future work should document and test these aspects of the prototype before a larger, controlled study evaluates sustained use, motivation, and motor outcomes over an appropriate rehabilitation period.

## Figures and Tables

**Table 1:** Design specifications of MIDAS.

**Hand Exoskeleton Subsystem**	
Weight	748 g
Force and motion transmission system	Linkage mechanism
Material	PLA 3D-printed rigid parts and Smooth-On Dragon Skin 20 silicon soft pads
Actuator	One MG-996R servomotor (Operating Voltage = +5V, Specified Stall Torque = 9.4 kg cm, Operating Speed = 0.17 s/60°, Rotation = 0–180°, Weight = 55 gm) to cause flexion and extension of subject’s fingers.
Securing method	Cord-lock design for securing on the fingers and double-sided Velcro straps for securing on the forearm and wrist
EMG sensors	Surface EMG sensors
**VR subsystem**	
VR headset	Oculus Quest 2
VR handheld control device	Provided with Oculus Quest 2
VR Game	Number of stages = 5, duration of each stage = 3 min, game environment scenes = natural location views for outdoor environment and earthy color palette for indoor environment, sonic effects = cheerful music, bird chirps, sound of mouse character, and pentatonic scales-high-pitched chimes
**Olfactory subsystem**	
COVID-19 preventive face shield	Curved PET frame
Scent	Mixture of lemon and bergamot scent
Mechanism of scent release	Piezoelectric transducer vibrating at ultrasonic frequency breaks the liquid scent into fine mist
**Control system**	
Microcontroller	Adafruit Feather nRF52832
Graphic display	SSD1306 128x32OLED
Voltage step-up module	MT3608 DC-DC step-up module
Power supply unit	2 × 3.7 V rechargeable Li-ion Panasonic NCR18650B batteries

**Table 2:** Demographic details of stroke patients ^1^.

Subject Number	Gender	Age (Years)	Level of Education	Marital Status	Impaired Side	Time Since Stroke Onset (Months)
**1**	Male	56	Between secondary and tertiary	Married	Left	38
**2**	Female	52	Tertiary	Single	Left	36
**3**	Female	27	Tertiary	Married	Right	17
**4**	Male	61	Tertiary	Married	Left	162
**5**	Male	46	Tertiary	Single	Right	114

^1^ Descriptions recorded during recruitment; no standardized stroke severity scale was recorded for these participants.

**Table 3:** User experience scoring system.

Score								
Obtrusive	O	O	O	O	O	O	O	Supportive
Complicated	O	O	O	O	O	O	O	Easy
Inefficient	O	O	O	O	O	O	O	Efficient
Confusing	O	O	O	O	O	O	O	Clear
Boring	O	O	O	O	O	O	O	Exciting
Uninteresting	O	O	O	O	O	O	O	Interesting
Conventional	O	O	O	O	O	O	O	Inventive
Usual	O	O	O	O	O	O	O	Leading edge

**Table 4:** SAM scoring system.

Scale	Valence Rating	Arousal Rating	Dominance Rating
**5**	Pleasant	Excited	Dependent
**4**	Pleased	Wide-awake	Powerlessness
**3**	Neutral	Neutral	Neutral
**2**	Unsatisfied	Dull	Powerful
**1**	Unpleasant	Calm	Independent

**Table 5:** Response of the subjects to the user experience questionnaire.

	CD ^2^	D ^2^	SD ^2^	NAD ^2^	SA ^2^	A ^2^	CA ^2^
**Item**	1st/3rd	1st/3rd	1st/3rd	1st/3rd	1st/3rd	1st/3rd	1st/3rd
Supportive	0/0	0/0	0/0	2/0	1/1	2/3	0/1
Easy	0/0	0/0	4/1	0/1	0/0	1/2	0/1
Efficient	0/0	0/0	2/1	1/1	2/2	0/0	0/1
Clear	0/0	0/0	1/0	1/1	2/1	1/2	0/1
Exciting	0/0	0/0	0/1	2/1	0/1	2/0	1/2
Interesting	0/0	0/0	0/0	1/1	0/2	2/0	2/2
Inventive	1/0	0/0	1/0	0/0	0/0	2/2	1/3
Leadingedge	0/0	1/0	0/0	0/1	1/0	2/3	1/1

^2^ CD = completely disagree; D = disagree; SD = slightly disagree; NAD = neither agree or disagree; SA = slightly agree; A = agree; CA = completely agree.

**Table 6:** Response of the subjects to the SRMS questionnaire.

Questions	CD ^3^	D ^3^	NAD ^3^	SWA ^3^	CA ^3^
If you have choices, do you feel like choosing to do rehabilitation using our device?	0/0	0/0	0/0	9/60	6/40
Do you feel like your motivation for participating in rehabilitation has gotten higher after trying our device?	0/0	0/0	4/27	6/40	5/33
Do you feel the device could be useful in helping you to exercise your hand?	0/0	0/0	3/20	5/33	7/47
Do you want to participate in rehabilitation with our device if you see other stroke patients getting better through rehabilitation?	0/0	0/0	1/7	8/53	6/40
Would you perform rehabilitation with our device even if no one is enforcing it on you?	0/0	0/0	1/7	7/47	7/47
Do you find participating in rehabilitation with our device exciting?	0/0	0/0	2/13	6/40	7/47
Does doing rehabilitation with our device help you feel like you are achieving something?	1/7	0/0	5/33	5/33	4/27

^3^ CD = completely disagree; D = disagree; NAD = neither agree or disagree; SWA = somewhat agree; CA = completely agree.

## Data Availability

The data presented in this study are not publicly available due to privacy and ethical restrictions, as they involve sensitive data collected from stroke-affected participants. Data supporting the findings of this study are available from the corresponding author upon reasonable request and with appropriate ethical approval.
